# Successful Cases Best Treated by Successful Remedies

**Published:** 1893-02

**Authors:** H. G. Blaine

**Affiliations:** Professor of Diseases of the Nervous System, Toledo Medical College


### Selections and Abstracts.

### SUCCESSFUL CASES BEST TREATED BY SUCCESSFUL REMEDIES.

BY H. G. BLAINE, A. M., M. D.,
Professor of Diseases of the Nervous System, Toledo Medical College.

Modern books have in a measure omitted from the classification of drugs a class which formerly went by the name of alteratives, and which were of so much importance that a physician who has been in practice for a number of years cannot resist the temptation to yet speak of remedies having such action as being actually alteratives. These are vegetable and mineral. The former particularly have always been in great demand in a number of cases where the natural tendency of the disease is to produce wasting and debility. Scrofula, a term by which we formerly meant a tubercular affection other than of the lungs, and at present a tubercular disease of the glands, bones and joints, is one of the diseases in which this class of remedies is decidedly applicable. In the post-tertiary stage of syphilis they also frequently act so nicely that we must continue to give them the preference over minerals. Recently there came to our notice a combination of such vegetable altertives, sold under the name, verrhus clemiana, and consisting of the fluid extracts of clematis erecta, prinos verticillatus, fraxinus americana and rhus glabrum, with one-eighth of one per cent. of venanatic acid, which has given such uniformly satisfactory results in these cases that we are tempted to recommend it as an alterative par excellence. It seems to possess the peculiar property of repairing wasted and disorganized tissues to a greater extent than any other preparation we have yet tried. The following are a few cases in which the preparation has given good results in my practice.

Chas. G., an Arabian, aged 36. Diagnosis, syphilis. Was under treatment at various places in this country. Came to me for treatment with secondary symptoms of the disease. Mercury did not seem to be borne well, and as it was at the time I first began useing verrhus clemiana I ordered a bottle, with directions to take a teaspoonful every four hours. Improvement was soon noticed. He continued to take the preparation till about one month ago, when he sailed for his native country with a bottle of verrhus clemiana and in excellent health and spirits.

Mr. P.—Strumous diathesis. Contracted gonorrhea about one and one-half years ago. After the usual treatment the discharge lessened, but would not cease entirely. In spite of all local treatment a gleety discharge continued. Finally I concluded to give him constitutional treatment with a view of correcting the tendency to struma, when, without any further local treatment, the discharge stopped. For the purpose of remedying the constitutional taint, I ordered verrhus clemiana. I do not pretend to say that lt is an infallible remedy for gleet, but in those cases in which the system is run down, or where there is a constitutional diathesis, I believe the cure will be greatly facilitated by giving this preparation.

Lillie B., age 5, came under my care one year ago. No history of phthisis in the family. She had measles when two years of age, and has continued weak ever since, and of late has developed a scrofulous aspect. From time to time eruptions made their appearance about the head and neck, and slightly on the trunk and lower extremities. They were at first pale, but gradually became red, and consisted of flattened elevations from about one-sixth to a fourth of an inch in diameter that were surmounted by a scab. In the early part of the year these spots took on inflammatory action and began to suppurate to the great annoyance of the patient. The patient began to grow weaker and weaker. This case I think must be looked upon as essentially scrofulous. The treatment which I adopted consisted of a liberal diet and verrhus clemiana. My patient improved rapidly under this treatment, and to-day there is no visible taint of any scrofulous affection. I look upon the above remedy as having effected a complete cure in this case, after all other remedies had been tried without effect.—*Ex.*

